# Female migrant domestic worker experiences during the COVID-19 pandemic in Hong Kong: A qualitative study

**DOI:** 10.1371/journal.pgph.0005399

**Published:** 2025-11-07

**Authors:** Timothy S. Sumerlin, Jean H. Kim, Roger Y. Chung

**Affiliations:** 1 The Jockey Club School of Public Health and Primary Care, The Chinese University of Hong Kong, Hong Kong SAR, China; 2 Center for Global Health Equity, NYU Shanghai, Shanghai, China; 3 Centre for Bioethics, The Chinese University of Hong Kong, Hong Kong SAR, China; 4 Institute of Health Equity, The Chinese University of Hong Kong, Hong Kong SAR, China; St John's Medical College, INDIA

## Abstract

During the COVID-19 pandemic, international migrant workers were often at risk of increased hardships due to disrupted employment, insecure residency status, and stringent government pandemic policies. Migrant domestic workers (MDW) who are typically required to live in the home of their employer, away from social support networks, may face additional stressors and vulnerabilities. This study seeks to understand the impact of the COVID-19 pandemic on female MDWs in Hong Kong. Semi-structured, one-on-one interviews were conducted from June 2021 to May 2022 with 20 female MDWs currently employed in Hong Kong. The study explored the impact of the COVID-19 pandemic on the MDWs: employment conditions, economic situation, family and social networks, and personal health. Thematic analysis, informed by the Employment Conditions and Health Inequalities Framework, was conducted on the interview data. Participants reported negative changes to employment conditions including new job duties, longer working hours, and not receiving statutory days off. Participants also felt that government COVID-19 policies unfairly targeted MDWs. Although MDWs often reported tolerating the negative work-related impacts of the pandemic to provide for left-behind family, MDWs would commonly seek other employment to cope with their employer-related difficulties. Additional stressors faced by MDWs during the COVID-19 pandemic were largely attributable to existing regulations and pandemic-related MDW policies that limited their autonomy. Improving the well-being of MDWs, particularly during public health crises, necessitates regulatory reforms that include MDW mental health as a priority area.

## Introduction

### Background

Lockdowns and social distancing measures caused by the COVID-19 pandemic created unprecedented global disruptions, amplifying existing social and economic inequalities [1,2]. International migrant workers, who numbered 169 million globally in 2019, are one of the populations disproportionately affected by the COVID-19 pandemic [3]. Working abroad to send remittances to left-behind family members, migrant workers often face inadequacies in healthcare access, impermanent residency status, low pay, and distance from social support networks [2,4]. As the pandemic progressed, migrant workers often experienced mental health distress from job loss, reduced wages, and heightened discrimination [4,5]. Moreover, these workers were often stranded in their host country due to border lockdowns. However, international migrant workers are an extremely heterogenous population, requiring separate examination of the working conditions and stressors of each occupational group.

### Migrant domestic workers

Female migrant domestic workers (MDW) constitute a significant workforce of 8.5 million people worldwide [6]. MDWs typically perform essential household services for their employer including cooking and cleaning, and often provide care for children, older adults, or people with disabilities. MDWs’ legal status in the host country is often linked to employment with the specific family that employs them, and so their ability to continue working abroad is often contingent on good standing with their current employer. In 2011, the International Labour Organization (ILO) adopted the Domestic Workers Convention (2011) (known as C189), an international treaty which sought to ensure MDWs with decent work, freedom of association, and a right to collective bargaining [7]. However, only 20% of ILO member states have ratified C189, none of which are major MDW destination countries. Hence, the rights afforded to MDWs differ across countries and regions, and MDWs may be at risk of harsh working conditions and other exploitations. For example, in the regions that most often employ MDWs, such as the Middle East and East Asia, labor policies require MDWs to live in their employer’s home [8–10]. This live-in arrangement creates a distinct living environment compared to other types of migrant laborer who may reside in dormitory facilities.

As a population heavily affected by social determinants of health, MDWs globally often face similar challenges – regardless of regional linguistic, religious, or cultural differences – including poor living and working conditions, limited healthcare access, insufficient financial and social resources, and inadequate policy protections [11–14]. Moreover, as an occupation which is largely comprised of women from the global south, who are often of different ethnicity from their host population and carry a lower social status based on their working conditions, lower pay, and temporary migration status, MDWs also face additional stressors from discrimination while abroad [11,15–18]. The COVID-19 pandemic, with its associated lockdowns and stay-at-home orders, introduced additional complexities for MDWs and their live-in working arrangement with researchers and rights groups calling attention to inadequate policy responses to protect MDWs [19].

### Migrant domestic workers and the COVID-19 pandemic

Dating back to the SARS epidemic in 2003, MDWs in Taiwan were noted to experience the outbreak and response differently from Taiwanese citizens with many MDWs reporting intensified discrimination and confinement in their workplaces [20]. In the COVID-19 period, research spanning from South Africa [21,22], the Middle East [23], North America [24,25], East Asia [26–31], and India [32] have indicated pandemic-related mental health problems [30,31], pandemic-related discrimination [15], worsening employer interactions with increased workloads [21,28,32], limited mobility [26,32], and barriers to COVID-19 vaccine uptake [33]. Collectively, these studies suggest failures in policies and inadequate social protection of MDWs which could result in financial losses, job loss, and poor health. However, most of these studies took place in 2020 at the early stages of the pandemic and may have missed the longer term effects of the protracted pandemic, especially as MDW-specific pandemic policies were introduced or evolved over time. Moreover, most previous literature looked at specific topics such as vaccine uptake or immobility but not a multi-dimensional viewpoint. As MDWs were likely affected by the pandemic at multiple levels, which are often interrelated and can intersect and impact one another, a “social determinants of health” perspective could provide a more encompassing understanding of the pandemic’s impact on MDWs.

### Study setting and COVID-19 context

The current study took place in Hong Kong SAR, China. Hong Kong has a population of 7.4 million, of which, approximately 340,000 were female MDWs in 2021, the year data collection began for this study [34,35]. Demand for MDWs in Hong Kong has been growing since the 1970s and is in part due to a large percentage of women entering the workforce, creating dual-earning households and requiring additional help with domestic work and caretaking of children and older adults. In 2023, the most recent year of available data, there were 356,231 MDWs in Hong Kong [35]. In Hong Kong, all MDWs must enter Hong Kong on a MDW work visa scheme which is sponsored by their employer. Under this scheme, all MDWs are required to live in their employer’s home (i.e., the live-in rule) and MDWs cannot legally partake in paid work outside the home of their employer [8]. If an MDW has not secured new employment within two weeks of completion or termination of her contract, she must repatriate to her home country (i.e., the two-week rule) [36]. MDWs are provided health insurance by their employer in case of physical illness, and they are given one mandatory day off per week (usually on Sunday). However, there is no statutory limit on weekly working hours for MDWs, which were reported to average 74–92 hours per week [37].

**Fig 1:**
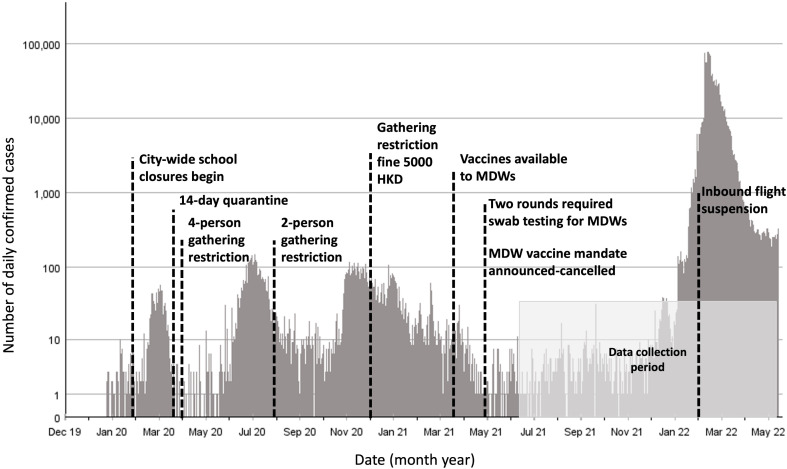
Timeline of key COVID-19 policies and events for migrant domestic workers with daily confirmed case counts.

Hong Kong maintained a relatively low incidence of COVID-19 infections in the first two years of the pandemic. During this time, Hong Kong included social distancing, mandatory mask wearing, rolling work from home recommendations, suspension of face-to-face classes for students, mass testing and contact tracing measures [38]. However, in early 2022, Hong Kong experienced its first sustained outbreak [39]. In addition to the population-wide pandemic prevention measures, MDWs in Hong Kong were required to comply with additional infection prevention measures including multiple rounds of mandatory swab testing, a short-lived COVID-19 vaccine mandate, increased scrutiny on changing employers, and quarantine measures which essentially prevented MDWs from leaving or entering Hong Kong for extended periods of time (see Fig 1 for timeline) [38,40–42].

### Study rationale and aims

The current study seeks to take a “social determinants of health” approach to explore the experiences of female MDWs in the mid to late stages (June 2021 to May 2022) of the COVID-19 pandemic in Hong Kong. Hong Kong, with its relatively large MDW population, strict pandemic prevention measures, and policies such as the live-in rule and lack of maximum weekly working hours—common to other host countries—provides an ideal context to examine the pandemic’s impact on MDWs. Findings from this study can provide nuanced perspectives by migrant workers on the effect that MDW-specific and pandemic-related policy measures had on multiple factors across the social determinants of health model. As Hong Kong’s MDW policies mirror those in many other host countries, by highlighting the lived experiences of MDWs, this study contributes to the evidence base needed for targeted policy reform and global public health strategies that address the unique challenges of migrant worker populations globally. Specifically, this study aims to:

1. Explore how the COVID-19 pandemic influenced MDWs’ employment conditions, economic situation, family and social networks, and personal health; and
2. Understand how MDWs reacted and responded to the changes related to the pandemic on their lives.

## Methods

### Ethics statement

Ethics approval was obtained by the Survey and Behavioural Research Ethics Committee of The Chinese University of Hong Kong (Ref. No. SBRE-20–584). Written informed consent was obtained by all participants prior to beginning each interview. Interview audio files were stored on a secure server only accessible to the researchers involved in the study. After transcription of all interviews and assessment for transcription accuracy, all audio files were permanently deleted. Written informed consent forms were stored in a secure locker at the university and were destroyed after completion of all study procedures. Interview transcripts (see Supplemental Material) were deidentified and participant names were removed. To ensure study quality, the consolidated criteria for reporting qualitative studies (COREQ) 32-item checklist was followed [43].

### Study design

This qualitative study employed in-depth, one-on-one semi-structured interviews to explore the experiences of MDWs in Hong Kong during the COVID-19 pandemic. The study was informed by an adapted version of the Employment Conditions and Health Inequalities Framework [44], which situates employment conditions as a critical determinant of health. Thematic analysis was conducted to identify key themes related to MDWs’ employment conditions, economic situation, family and social networks, and ultimately their health.

### Data collection

Participants were first purposively sampled from a prior MDW survey’s database, selecting those who had agreed to future research participation (n = 14). This database contained a diverse sample of MDWs with different employment histories, location of work residence, and background characteristics and participants were selected to reflect these differences [45]. Snowball sampling through participant referral was additionally used to obtain the remaining respondents (n = 6). A total of 20 participants were sampled from June 12, 2021, to May 15, 2022. Participants were contacted through messaging their private WhatsApp account with a number that they voluntarily provided with consent to be contacted and invited to join the study. Participants were not called at any point to protect them from needing to verbally discuss study participation while potentially in their employer’s home. Employers of study participants were not involved in the study and were not contacted or notified at any point. This was done to ensure participants felt freer to express their opinions on their working experience without employer influence. All interviewers were conducted face-to-face, and to further ensure participant privacy and confidentiality, the researchers allowed the participant to choose the time and place for the interview. Most interviews took place in public parks, which are popular gathering places for MDWs, and on the participant’s mandatory day off. No interviews were conducted in the employer’s home. Inclusion criteria included being a female from either the Philippines or Indonesia who was currently working as a MDW in Hong Kong for at least the past 3 months. Based on the interviewer’s language ability, proficiency in English was also required to participate in the study, which is the primary language used among MDWs during work in Hong Kong. Six potential participants could not be reached and only one refused to participate. After obtaining written informed consent, interviews were audio recorded, and field notes were taken. The average length of each interview was 45 minutes. Participants received a 200 HKD (approximately 25 USD) cash coupon for their participation. Interviews were conducted until the study authors agreed that no new themes were emerging, and data saturation was reached.

### Reflexivity statement

This study explores the lived experiences of female MDWs in Hong Kong during the COVID-19 pandemic. It was conducted by three Hong Kong-based researchers who had previous experience in qualitative research and prior work among the MDW population. We recognize that our higher educational attainment, distinct cultural and national background, and lack shared lived experience as MDWs could influence our perspectives and interactions with participants. To address these differences, the researcher responsible for recruitment and interviews (TSS, MPH, PhD candidate) underwent cultural sensitivity training tailored to engaging MDWs. We also ensured participants felt comfortable by allowing them to choose the timing and location of interviews—often held in public parks, a familiar gathering place for MDWs on their day off. Participants were also made aware of the research purpose and the interviewer’s positionality prior to conducting interviews. Although each researcher holds Hong Kong permanent residency status, they originally come from the United States and share the broader experience of migration to Hong Kong. Moreover, the COVID-19 pandemic introduced shared societal challenges, such as social distancing and border lockdowns, which informed our collective understanding of the research context. Supervisory roles were held by JHK (ScD, Associate Professor) and RYC (PhD, Associate Professor).

### Semi-structured interview guide

A semi-structured interview guide was designed based on the Employment Conditions and Health Inequalities Framework within the context of the COVID-19 pandemic. Interview questions covered the daily living and working experience of participants, their economic situation, their family and social networks, and their personal health during the pandemic. Interview questions were first pilot tested with a few MDWs and other researchers who were not involved in the study and then adjusted for better understanding before commencement of the interviews. Before beginning each interview, participants were assured that MDWs encounter a full range of experiences throughout their employment as an MDW, ranging from positive to negative, and interviews were only interested in their authentic experience as an MDW. Follow-up and probing questions were also asked, when needed.

### Data analysis

**Fig 2:**
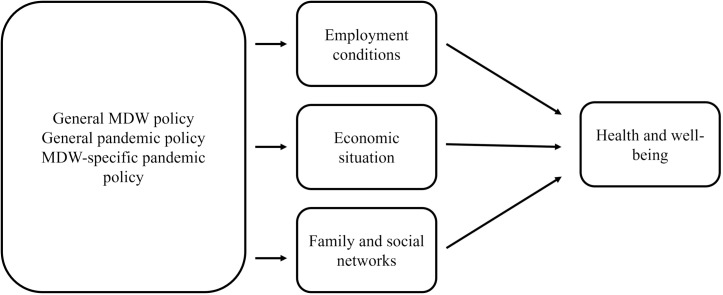
Analytical framework.

Audio recordings were transcribed verbatim by one of the authors. Thematic analysis was used based on the study’s analytical framework informed by the World Health Organization’s Employment Conditions and Health Inequalities Framework (see Fig 2) [44,46]. Interview transcripts were first thoroughly read by an author to become familiar with the content and themes were coded across interviews. Broad themes (pandemic effects of: employment conditions, economic situation, family and social networks, and personal health) were first deductively coded based on the analytical framework and subthemes were further inductively coded within each broad theme (see Table 1) [47]. The two additional authors evaluated the accuracy and consistency of the coding, and all authors came to an agreement before coding was finalized. All data was managed with NVivo 12 [48].

**Table 1:** Study themes, subthemes, and participants contributing to each theme.

Theme	Sub-theme	Participant contribution to theme/subtheme
Pandemic effects on employment conditions	(a) Workload changes and restrictions	P1, P3, P4, P5, P7, P8, P9, P11, P12, P14, P15, P16, P17, P18, P20
	(b) Workplace conflicts	P3, P4, P5, P8, P11, P12, P14, P16, P17, P18, P20
	(c) Stable working environment	P1, P6, P9, P10, P11, P13, P16, P20
	(d) Job mobility	P3, P4, P7, P11, P14, P15, P16, P18, P20
Pandemic effects on MDWs’ economic situation	(a) Remittance pressures	P1, P2, P4, P6, P7, P10, P11, P13, P17, P18, P19, P20
	(b) Savings and debt	P1, P10, P11, P17, P18
	(c) Compensation issues	P1, P9, P10, P14, P17, P18, P19
Pandemic effects on family and social networks	(a) Emotional impact of separation	P1, P2, P3, P4, P7, P8, P9, P10, P11, P14, P15, P19, P20
	(b) Social isolation	P1, P2, P4, P6, P7, P8, P10, P11, P12, P14, P15, P17, P18, P19, P20
	(c) Disconnected from religious practice	P1, P2, P4, P6, P7, P9, P13
	(d) Adapt to online religious practice	P3, P5, P12, P14, P17, P20
Pandemic effects on personal health	(a) Physical health	P12, P14, P16, P19
	(b) Mental health	P3, P4, P7, P8, P11, P14, P15, P16, P17, P18, P20
	(c) Employer influence	P3, P4, P14, P15, P18, P19, P20

## Results

Among 20 total participants, 19 were from the Philippines and one from Indonesia (median age = 36; median years working in Hong Kong = 4). Nearly half of participants were single, never married (n = 9), five were married, and six were separated. Nearly half reported secondary school as their highest educational attainment and five reported receiving a bachelor’s degree. Only one participant reported a monthly salary below the minimum statutory salary for MDWs at the time of the study (4,630 HKD = approximately 590 USD). Seven participants changed their employer at least once since January 2020.

### Pandemic effects on employment conditions

Table 1 displays the study’s themes, subthemes, and participants contributing to those themes. Prior to the COVID-19 pandemic, many MDWs reported poor living conditions (e.g., sleeping in the living room, no access to air conditioning, extremely restricted access to food). Several participants also reported work beyond their contract requirements (e.g., cleaning the homes of others without pay, personal body massages). However, participants claimed the pandemic made an already difficult working environment more challenging with changes to their workload, new job duties, and restrictions on movement. Most participants reported feeling exhausted from the greatly increased cleaning and sanitizing in the home during the first several months of the pandemic. Participants who believed their employer had a high expectation of the MDW’s cleanliness, but a low expectation of their own cleanliness, expressed frustration:

They [the employer] want you to clean it more. Everything you have to sanitize, the door, the food from the outside, and if you go inside, you have to clean your shoes or slipper…At first, it’s weird, like really insult me because why I’m the only one who has to do this? (P11)

Some participants felt their required work duties during the pandemic went beyond their tasks as MDWs. Tutoring children and monitoring their online class work was the most reported new job duty. Those MDWs expressed irritation in being required to tutor children for subjects they were not knowledgeable in, such as Chinese language, as one participant described:

But sometimes I’ll say, ‘why everything my fault?’ She should fix her son, you know, especially Chinese [class]. I said, ‘you cannot depend on me because I’m not Chinese. Of course, sometimes I don’t know how to write Chinese character properly.’ I’m lucky if I only get [yelled at] three times a week. (P04)

Restriction in movement was frequently discussed as participants were often required to remain within the confines of the home on their day off and many were required to work while at home. Those who were paid extra (day off pay ranged from 150 to 300 HKD, equivalent to 19–38 USD) found this to be advantageous, while for those paid nothing, it was seen as an increased stressor. Others who were required to take their rest day at home reported having no place to rest and could only lay on their bed or stand in the kitchen.

Interpersonal conflict between participants and members of the employer’s family was a consistent theme that was usually attributed to a more intense and cramped working environment. Many participants reported increasing incidence of being yelled at by their employer, resulting in heightened nervousness and stress. Conflict also arose through participant’s belief that their work was more closely watched and unfairly criticized when family members remained home. One participant described always needing to appear busy:

It’s pressure, you just stay in the kitchen. You cannot go outside because they’re working, and the parents are working in the outside and then other ones in the bedroom… They want to see you always busy. (P14)

In contrast to the difficulties reported above, some participants reported stable working environments with clear working hours, guaranteed full 24-hour rest day per week, and adequate living space. They also reported feeling trusted by their employer and feeling that they had freedom and agency in their job.

Participants reported different ways of dealing with difficulties in their employment conditions, with the opportunity for job mobility often being discussed. However, due to the pandemic prevention measures in Hong Kong (e.g., quarantine requirements), several participants felt they had no choice but to tolerate these conditions for fear of job loss and deportation. Participants experiencing workplace stressors believed their employers took advantage of the current MDW policies and pandemic-specific policies, knowing MDWs had limited options. Despite this, to address the above challenges, half of the interviewed MDWs changed to a new employer when their contract expired or had planned to do so.

### Pandemic effects on MDW’s economic situation

The main source of economic impact from the pandemic was the increased pressure to send remittances to left-behind family. Several of the participants reported increasing the frequency and amount of money sent home due to pandemic-related job loss of family members. Four participants reported that their financial burden was further amplified by a weakened exchange rate, as the Hong Kong dollar depreciated against the Philippine peso, requiring them to send larger remittances to support their families. Despite the increase in the remittance amount sent home, participants emphasized that providing financial support to their family was their priority in taking a job as a MDW initially, and they were glad to help.

Decreased financial savings and even debt stemming from the pandemic were reported by several participants. One source of decreased savings came from increased remittances home while another source of unexpected expenditures was through the purchase of personal protective equipment like masks and sanitizer. While many employers provided these items to their MDW for free, those who were required to pay on their own complained that it created a financial strain. One participant even described needing to take out a loan from the bank to cover the increased financial demand from her family and to pay for the unexpected costs of personal protective equipment, which were not covered by her employer.

The pandemic resulted in instances of both compensation gain and loss for participants. In accordance with their employment contract, MDWs are legally entitled to two weeks holiday and a paid airfare ticket home after completion of their contract. Due to pandemic travel restrictions, MDWs had to negotiate solutions to lost annual leave and an inability to fly home. Some participants reported continuing to work during their holiday and being paid extra while others also worked for free and were verbally told they would get extra time off in the future, when pandemic prevention measures ended. While most participants were provided the cash equivalent of their flight ticket, one participant reported that her ex-employer, who was upset the participant did not renew the contract, provided an actual ticket home, denying her the cash equivalent of the ticket. As mentioned in the pandemic effects on employment condition’s theme, participants who were restricted from leaving the home on their day off were often required to continue working. Those who were compensated for this extra work welcomed the arrangement, while those being required to work with no additional pay viewed it as a financial loss.

### Pandemic effects on family and social networks

Most participants reported missing their left-behind family due to an inability to visit home because of pandemic restrictions on foreign travel and blamed stringent quarantine policies on their inability to visit home. Many participants felt the policies often changed which increased their uncertainty as to whether they could return to Hong Kong after a visit to their home country:

I think here in Hong Kong is getting worse…because of the policy. Sometimes they are changing the policy...Sometimes we want to go back in the Philippines for vacation, but we are afraid if the policy will change again, then they ban again the flight. (P15)

Most participants were also worried for the health of their family, especially their parents and children. However, those with family living in rural areas of their home country felt less concerned for their health. Frequent texting, phone, and video calls were used to communicate with family daily.

Reported missed major family events included the loss of a close family friend from COVID-19 infection and the loss of a parent. Two participants had a parent pass away while they were working in Hong Kong but were unable to return home due to travel restrictions and hesitant employers. The participant whose mother passed away described the conflict with her employer:

She asked me if I want to go back and see my mother for the last time. I asked, ‘Is it ok for you?’ But she said, ‘if you go back now, I will take another helper.’...It’s more painful, it’s like I was brokenhearted two times. (P14)

Social isolation was reported by 75% of participants who reported downsizing their social circles to one or two friends or limiting their social interaction to a relative also working in Hong Kong. Throughout the COVID-19 pandemic in Hong Kong, gatherings in public spaces were often limited to a maximum of two or four people. Several participants said they stopped gathering with friends because they feared police monitoring in public spaces and worried about receiving fines. While some participants already preferred to spend their day off alone, the feeling of most participants is summarized below:

Before we can go wherever we want to go...But now, you see few places can go. And some of my friends cannot go out. Their employer does not allow them to go out…Also, on the rainy days you cannot sit over there, over there, because have so many police. We are so scared. (P17)

All participants identified as either Roman Catholic or Protestant Christian, however, the pandemic’s effect on their church attendance was mixed. Due to pandemic policies, church services were periodically moved to online platforms. Some participants found this as an opportune time to quit attending altogether. Other participants attended online and reported feeling closely connected with their church community and God. For those who found religious service attendance to be important, prayer was emphasized for coping and stress reduction. When in-person attendance was permitted, most participants were hesitant to return, fearing the crowded environment. As one participant put it, “I will go to the real church, to pray, that’s okay. But if I will go to maybe chapel or stadium and they just sing, I don’t know, you’re just too crowded.” (P02)

### Pandemic effects on personal health

Only one of the 20 interview participants had been infected with the COVID-19 virus at the time each interview took place. This is in part due to the relatively low transmission rates of COVID-19 in Hong Kong up until early 2022. One participant reported experiencing weight loss due to stress in her working environment. Two participants described their experience with pre-existing chronic conditions. Although they reported an increased sense of personal responsibility to prevent infection, both had difficulty maintaining adequate medication supplies due to fear of infection at the hospital and employer intervention. When asked whether she had difficulties obtaining her medication, one participant explained:

The last time my employer didn’t allow me to go outside. So, I asked my mom [female employer] if I can go out a bit to get the medication, because the doctor said I cannot stop the medication…My employer booked me the taxi, because she wouldn’t let me bus or MTR (subway).” (P19)

In comparison to physical health effects, the pandemic’s effect on mental health was more pronounced by participants. Heightened stress was reported by half of the participants and was largely attributed to the increased workload and lack of privacy in the workplace. Sadness and loneliness were attributed to extended separation from family members and social isolation, though contacting family and friends through phone calls and messaging was reported as a useful coping mechanism.

Employers were often involved in their MDW’s pandemic health decision-making. This was seen in provisions of personal protective equipment and influence over COVID-19 vaccination. Several participants felt employers had expectations of MDWs exceeding the employer’s own protective behaviors, as one participant described:

She [female employer] doesn’t wear a face shield. So...why I’m wearing this thing, and you’re just wearing a single mask, but I need to wear two masks when I go out? Like two masks and a face shield, it’s kind of weird. (P18)

Some participants explained their employer tried to prevent them from being vaccinated in case side effects lowered their productivity or for fear of a financial burden in case of death. Others described being forced to vaccinate, being told they could not have a day off or would lose their job if unvaccinated. Others reported methods to push vaccination included being told vaccine supplies were quickly dwindling or they could easily travel back home once vaccinated. In response to the two rounds of mandatory swab testing for all MDWs in May and June 2021, participants came to a consensus that it was good for their health, but unfair to be targeted and singled out from the general population.

## Discussion

This qualitative study explored the experiences of female MDWs in Hong Kong in the latter stages of the COVID-19 pandemic. Using a social determinants of health framework, we found participants were required to take up new job duties, experienced increased tensions in their employer’s home, took financial losses, and faced extended separation from family and social isolation. These factors are largely attributable to inadequate protections given to MDWs in their work contracts and pandemic policies which disproportionately affected migrant workers in Hong Kong. These themes also interacted with each other with compounding effects often resulting in worsening mental health. The findings from this study provide important insight of the lived experience of these migrant workers with implications to improve MDW policy and MDW well-being.

### Consideration of past findings and new findings

The findings of this study confirmed many of the findings from studies of MDWs in Hong Kong [26–28] and abroad [21–25,29,31]. Previous studies have also noted the stress induced from increased workload [21,22,28] and conflict between MDW and employer [28], which were likely exacerbated by pandemic policies that limited mobility outside of the home [19,24,26,32]. Many study participants reported social exclusion, experiencing differential treatment from their employer’s family at home (e.g., additional sanitation practices when entering the home) and feeling unfairly targeted by police in public spaces, which restricted their full participation in both settings. This likely worsened the mental health challenges MDWs already faced due to their demanding work environment. A 2020 study found relatively high rates of probable anxiety (31.8%) and depression (26.9%) among Indonesian MDWs in Macao, Hong Kong, and Taiwan early in the pandemic [31]. Protective factors of anxiety and depression from this study included MDWs who were older, married, had higher incomes, higher educational attainment, and had better language proficiency in English and Chinese [31]. Our findings and past findings highlight the heterogeneity and intersectionality within the MDW population. Rather than being a monolithic group, MDWs differ in their nationality, age, education level, and family situation while also taking the role of an MDW, a female, a migrant, and a provider for their left-behind family in their home countries. All these factors may interact and influence each other, affecting MDW health differently. In our study, marital status most clearly influenced MDWs’ pandemic experiences. Married participants or those with children generally faced a greater burden to send extra remittances, while single participants without children reported less burden. However, participants with a burden to send extra remittances also expressed pride and delight in doing so. In the current study, the participant’s age, education level, and years spent working as a MDW in Hong Kong did not show any consistent relationships with differing pandemic experiences, while inference on nationality is limited due to 19 of 20 participants being Filipino.

Several study participants reported working conditions aligning with the ILO’s forced labor indicators, potentially meeting the ILO’s modern slavery definition: “work which is exacted from any person under the menace of any penalty and without voluntarily consent” [49,50]. Forced labor indicators described in this study included abusive living and working conditions, excessive overtime, physical and sexual harassment (i.e., required massages), restriction of movement, isolation, and intimidation and threats. While a single indicator may not constitute forced labor, a combination or severity of a condition may indicate so. Moreover, some indicators are also violations within Hong Kong law, such as sexual harassment, and indicate that greater surveillance and reporting mechanisms are needed to adequately address these violations. However, other violations, such as excessive overtime, are largely permissible within current Hong Kong law as there is no limit to weekly working hours. Yet, violations to participants’ mandatory weekly 24-hours off was frequently reported during the pandemic, and even if extra payment was made, this would constitute excessive working hours by violation of the mandatory one day off rule.

While discussions about MDWs often focus on their “invisibility” [9,27], “violence” [22], and “discrimination” [15], our study also emphasizes their agency during a public health crisis. There are well-documented power imbalances between MDWs and their employer—evident in this study and previous studies [28,32,51], as many MDWs feel that policies favor employers, forcing them to endure hardships to maintain their jobs. The pandemic exacerbated these challenges, with other studies noting that job losses increased MDWs’ risk of homelessness or returning to their home countries without work [21,28,29,52]. However, our interviews with currently employed MDWs revealed a more nuanced picture: although many faced overwork. Unfair treatment, and restricted movement early in the pandemic, those who changed jobs reported improved, more stable conditions. Moreover, MDWs demonstrated empowered by migrating abroad to earn significantly higher wages and support their families back at home, a role they valued despite the stress of increased remittance demands.

This study further highlights the transitory nature of a continually evolving public health crisis, like the COVID-19 pandemic. Early in the pandemic, excessive cleaning was a key issue, while later stages revealed the importance of addressing social isolation and creating job mobility to improve employment conditions and well-being. Importantly, most study participants experienced significant disruptions from pandemic policies and their impact on employment and well-being, despite not being directly infected by the virus. These findings underscore the prolonged mental health consequences driven by social determinants of health throughout all stages of the pandemic.

### Effect from policy and policy interventions

The exploitation of MDWs in Hong Kong must be understood within the broader framework of gendered labor, capitalism, and migration policies. Domestic work is a highly feminized occupation [6], with women disproportionately filling these roles due to long-standing gender norms that associate them with caregiving. Under neoliberal economic models, like those in Hong Kong, MDWs are a critical yet undervalued labor force, sustaining middle- and upper-middle class households while remaining structurally disadvantaged. Current policies in Hong Kong, such as the live-in rule, further reinforce a system of labor dependency, leaving MDWs with little bargaining power and at risk of exploitation. Without adequate legal protections, like those afforded in C189, MDWs often experienced excessive work hours, restricted mobility, and workplace abuse. These conditions were magnified during the COVID-19 pandemic, highlighting the need for stronger legal protections. While ratification of C189 is likely to remain low, regions that depend on MDWs, like Hong Kong, may consider amending local labor policies to more closely align with its recommendations.

During the pandemic, Hong Kong implemented MDW-specific policies including mandatory COVID-19 testing, a short-lived vaccine requirement, designated quarantine hotels, and strict gathering limits [41,42]. Similar policies affecting MDWs were present globally [19]. While these policies were not inherently unreasonable, many MDWs believed it was unfair when they were specifically targeted by these policies. In the formation of the MDW-specific COVID-19 policies, there was a noteworthy lack of input and consultation of MDWs. The lack of political voice and limited advocacy in the formation of MDW labor policies is an important area for redress for improving the working conditions and mental health of these workers worldwide. Furthermore, many employers in this study were found to intervene in the health decision-making of MDWs in both COVID-19 and non-COVID-19 health issues. Employer involvement in MDW health decision-making has been found globally in studies from Canada [24], South Africa [21], Malaysia [53,54], Singapore [55], India [32], and Hong Kong and Macao [33,56,57]. As an issue affecting MDWs globally, interventions to improve MDW health literacy and healthcare decision-making autonomy is needed to better empower MDWs.

In countries around the globe, while border closures and social distancing affected all residents, MDW’s were at much higher-risk of social isolation and inadequate social protection due to government policies and lack of provisions for MDWs [19]. A policy analysis from Taiwan found policymakers inadequately considered migrant workers in their emergency measures and restrictions and were additionally viewed as potential sources of infection when discussed [58]. In Canada, MDW applications for permanent resident status nearly stopped which left MDWs in a temporary migrant status with limited mobility [24]. In Lebanon and Saudi Arabia, MDWs reported homelessness after losing their jobs and being unable to return home [52]. MDWs in Singapore attributed open communication between MDW and employer and social support networks as crucial to their well-being, while social isolation during the pandemic was a contributor to their worsening mental well-being [29]. Social distancing restrictions and police enforcement led to increased isolation among MDWs, potentially weakening crucial social and religious networks, which will require post-pandemic assessment for long-term impact [59,60]. In Hong Kong, MDW-specific quarantine hotels created additional barriers due to limited space that was allocated on a first come, first served bases which likely contributed to labor shortages. Policy inconsistencies caused anxiety, particularly during family emergencies. Future pandemic responses should consider emergency home visit provisions through the immigration department.

There are several policy implications of this study. First, to address the policy shortfalls described, a health-centered approach to policy should be incorporated into migrant worker schemes. Health in All Policies (HiAP) recognizes health beyond the health sector alone and believes health outcomes primarily stem from broader policies affecting the population [61], which is also reflective of the concept of “social determinants of health.” A HiAP approach would look beyond the economic impact currently driving MDW policy and consider how policies may prevent and minimize health disparities, empower MDWs, and create healthy and safe environments. For example, the pandemic highlighted key issues in the mandatory live-in rule which was enacted in Hong Kong in 2003. Despite advocacy efforts for a live-out option [62], this requirement persists, potentially exposing MDWs to longer working hours and abuse by living around the clock with their employer [37,63]. Living separately may protect MDWs’ privacy and prevent forced work on rest days, subsequently improving their well-being.

Worldwide, the autonomy of MDWs is often constrained by restrictive work schemes that increase their dependency on a single employer and reduce their ability to negotiate fair working conditions. To improve the autonomy of MDWs, countries should allow MDWs to work part-time for more than one employer. In addition to protecting MDWs from unreasonable working conditions due to dependency on a single employer, this arrangement could also be favorable to families who cannot retain a MDW full-time. Countries such as Singapore, which employs 268,500 MDWs, in 2017 commenced a working scheme by which domestic helpers are hired by a company rather than a single household [64]. Under this scheme, MDWs reside in company housing instead of a family’s home and work for multiple households providing specific services like home cleaning, shopping and cooking, child-care, and elder care [65]. Implementing a program like this in other countries and regions could also simultaneously pilot test live-out facilities for MDWs who would live in company housing. However, barriers to developing and implementing such policies elsewhere must consider the context of each locality. For example, in Hong Kong, to enact a live-out option and part-time worker scheme, land would likely need to be allocated to build migrant worker dormitories which may lack support from the public due to affordable housing shortages already present in the city. Additionally, in regions such as Hong Kong, the live-in rule was established to prevent illegal part-time work. Therefore, in order to implement a working scheme that allowed for MDWs to work for more than one household, additional policy and surveillance measures would be needed to prevent and detect illegal part-time work. All these policy changes would require significant legislative action and political will.

### Non-policy interventions

Interventions which can take place in the shorter term should also be considered since changes to policy often require extended time. These interventions should consider cost, barriers, and feasibility of implementation to be across the MDW population. To directly address mental health challenges that MDWs face, which are linked to their working environment and exacerbated by the COVID-19 pandemic [11,15,45], digital mental health interventions, such as the WHO’s Step-by-Step app, may be considered [66]. This tool uses a guided, technology supported, intervention for those with depression and was created to address treatment gaps in mental health, particularly among those from lower- and middle-income countries [66]. Previously, Filipino MDWs in Macao showed an overall positive response to the use of digital mental health tools [67], and the Step-by-Step app has been culturally adapted and evaluated for its effectiveness among this same population [68–70]. This digital tool provides free or low-cost, discreet mental health support through a personal phone, and addresses MDWs’ time constraints while avoiding potential stigma from their employer. In addition to seeking care individually, in the post-COVID-19 era, a continued effort should be made to support and rebuild MDW support groups as social isolation during the pandemic was often discussed as an issue. Governments may consider providing additional financial resources to local charities and organizations focused on MDWs. For example, in Hong Kong, HELP for Domestic Workers is a local organization that provides advice, assistance, education, and some counseling services to MDWs [71]. Providing additional financial assistance and resources to organizations that are already familiar with the MDW population, and their needs, could be a way to more effectively and efficiently build MDW local support systems.

### Limitations and future research

Some limitations must be considered with the study’s findings. First, pandemic prevention measures which often included limitations to public gatherings to just two to four people complicated the data collection process. Second, the inherent smaller sample size and non-probability sampling that is employed in qualitative research may limit the generalizability of the study’s findings to the MDW target population. However, qualitative research provides rich data and nuanced answers to research questions that are often unattainable in quantitative studies. Sampling from a previous study’s database and referrals from study participants may have biased results to a particular subset of experiences based on the source of sampling; however, our participants were spread out across Hong Kong, with employers from differing nationalities and socioeconomic levels. Our sample’s working history in Hong Kong was also diverse, ranging from one to 17 years. Third, all interviews were conducted in English, the primary language used between MDWs and employers. However, MDWs who do not speak English well, particularly Indonesian MDWs, may not have participated in the study due to language barriers. This limitation may have skewed the study’s results towards MDWs who were more highly educated, with higher English proficiency, and Filipino. MDWs who do not speak English often also have limited Cantonese language proficiency, the Chinese language most spoken in Hong Kong. MDWs who have limited verbal communication with their employer likely faced even more challenges throughout the pandemic which underscores the need to address the issues found within the current study. Our study sample contained only one Indonesian MDW. This likely limited the perspective Muslim MDWs, most of whom are Indonesian, who may have had differing experience from their Catholic and Protestant counterparts. Fourth, as this study only sought the MDW perspective, the potentially differing perspectives of other stakeholders including employers, policymakers, and MDW advocates were absent. This should be addressed in subsequent research. The study also conducted interviews over an 11-month period during the pandemic and each of these MDWs were interviewed in a single time point and not followed long-term to assess changes to their working conditions as the pandemic restrictions eased. Past studies from Hong Kong have shown that population perceptions of susceptibility, levels of infection-related anxiety and disease prevention behaviors evolve greatly as pandemics evolve [72,73]. Yet, this study covered the experiences of MDWs ranging from 1.5 years to 2.5 years of the pandemic which provides useful insights into evolving working conditions during this period. Finally, as the study covered potentially sensitive topics, those facing particularly poor circumstances may have refused participation due to trauma or fear of repercussions by their employer. However, this suggests there are situations in even more need of policy changes to protect those facing exceptional circumstances.

Future studies may consider a quantitative approach to empirically assess the post-COVID-19 mental health and employment conditions of MDWs. To obtain a comprehensive understanding of employer household environments, studies may consider the employer experience during the pandemic. Future studies may also consider the employer’s perspective and knowledge of MDW-related policies to have a more comprehensive understanding of ways to approach policy change in the future. Finally, stakeholder interviews with MDW organizations, sending country policymakers, and host country policymakers to understand the feasibility and barriers to recommended policy changes from this study are warranted in the future to assess effective pathways for improving MDW well-being.

## Conclusion

This qualitative study from Hong Kong highlights how pre-existing structural inequities in migrant labor policies were amplified during a public health crises for MDWs. During this period, MDWs often experienced heightened job demands, prolonged working hours, and social isolation. These findings resonate with patterns observed across East Asia and globally, where similar policies have put MDWs at-risk of poor working conditions and poor health. These health concerns can be partly addressed through reforms that increase the autonomy of MDWs as well the implementation of mental health interventions for MDWs. MDW labor laws should allow for MDWs to exit from intolerable employment conditions without fear of deportation or significant financial loss. During the pandemic, MDWs were essential workers in many regions of the world, performing job duties such as caretaking of elderly and children. Safeguarding their well-being requires increased dialogue and input from MDWs in the formation of MDW labor policies.
