# Supplementary material for: Female migrant domestic worker experiences during the COVID-19 pandemic in Hong Kong: A qualitative study
Source: PLOS Glob Public Health. 2025 Nov 7;5(11):e0005399. doi: 10.1371/journal.pgph.0005399 (PMC12594351; doi:10.1371/journal.pgph.0005399)
Supplement: Attachment — Submitted filename: Response to Reviewers.docx [file pgph.0005399.s002.docx]

PLOS Global Public Health

PGPH-D-24-01388
Female migrant domestic worker experiences during the COVID-19 pandemic in Hong Kong: A qualitative study

Dear Dr. Nancy Angeline Gnanaselvam, Academic Editor,

Thank you for the opportunity to revise our manuscript. We are grateful for your careful reading of the revised version of our manuscript and hope that we were able to address all the comments and concerns in the following responses. In addition to this Response to Reviewers file, we have also attached a Track Changes version of our manuscript and a clean version of our revised manuscript.

We apologize for the delay as we waited for further guidance by the journal regarding data availability policies.

**Response to Editor Comments**

1. Table 1: There are possible identifiers which can be used to identify the participants. Kindly convert the table to a paragraph by only presenting the summarised findings of demography.

Thank you for this suggestion. We agree that removing Table 1 and converting it to a descriptive paragraph can help de-identify our study participants while maintaining all the relevant information for the readers. Our revised paragraph covering the contents of Table 1 is below, and Table 1 has been removed from the manuscript.

“Among 20 total participants, 19 were from the Philippines and one from Indonesia (median age = 36; median years working in Hong Kong = 4). Nearly half of participants were single, never married (n = 9), five were married, and six were separated. Nearly half reported secondary school as their highest educational attainment and five reported receiving a bachelor’s degree. Only one participant reported a monthly salary below the minimum statutory salary for MDWs at the time of the study (4,630 HKD = approximately 590 USD). Seven participants changed their employer at least once since January 2020.” (Results, paragraph 1, lines 183-189)

2. The attached transcripts have multiple possible identifiers. Hence, I recommend authors to just mention that data is available with investigators and the corresponding author can share the data on reasonable request. Please remove all identifiers such as past employment, age, languages, age of children.

We agree that there are multiple possible identifiers throughout the interview transcripts and that publicly sharing these transcripts may risk exposing the study participants given the study population’s size and location. To follow PLOS Data Availability policies, we have amended our Data Availability statement (below) and have removed the Supplemental file from our revised manuscript.

“Data Availability

Interview transcripts are not publicly available because participants did not consent to release their data beyond the researcher team. Given the presence of multiple indirect identifiers and the relatively small population of migrant domestic workers in the study setting, it is difficult to fully de-identify the data to ensure participant privacy. Requests for data access may be directed to the Survey and Behavioural Research Ethics Committee of The Chinese University of Hong Kong at: fssc02@cuhk.edu.hk.”

Thank you again for your time in reviewing our manuscript and providing comments to make improvements in this final stage.
