# Supplementary material for: Female migrant domestic worker experiences during the COVID-19 pandemic in Hong Kong: A qualitative study
Source: PLOS Glob Public Health. 2025 Nov 7;5(11):e0005399. doi: 10.1371/journal.pgph.0005399 (PMC12594351; doi:10.1371/journal.pgph.0005399)
Supplement: Peer review history

# Author comment: Author response to Decision Letter 0

**DOI:** 10.1371/journal.pgph.0005399.r001

1 Sep 2025

Attachment Submitted filename: Revised manuscript with Track Changes.docx

# Editor report: Decision Letter 0

**DOI:** 10.1371/journal.pgph.0005399.r002

**Authors:** Nancy Gnanaselvam

19 Sep 2025

PGPH-D-25-02509

Female migrant domestic worker experiences during the COVID-19 pandemic in Hong Kong: A qualitative study

PLOS Global Public Health

Dear Dr. Sumerlin,

Thank you for submitting your manuscript to PLOS Global Public Health. After careful consideration, we feel that it has merit but does not fully meet PLOS Global Public Health’s publication criteria as it currently stands. Therefore, we invite you to submit a revised version of the manuscript that addresses the points raised during the review process.

Please submit your revised manuscript by Oct 19 2025 11:59PM. If you will need more time than this to complete your revisions, please reply to this message or contact the journal office at globalpubhealth@plos.org. When you're ready to submit your revision, log on to https://www.editorialmanager.com/pgph/ and select the 'Submissions Needing Revision' folder to locate your manuscript file.

Please include the following items when submitting your revised manuscript:

- A rebuttal letter that responds to each point raised by the editor and reviewer(s). You should upload this letter as a separate file labeled 'Response to Reviewers'.
- A marked-up copy of your manuscript that highlights changes made to the original version. You should upload this as a separate file labeled 'Revised Manuscript with Track Changes'.
- An unmarked version of your revised paper without tracked changes. You should upload this as a separate file labeled 'Manuscript'.

Guidelines for resubmitting your figure files are available below the reviewer comments at the end of this letter.

We look forward to receiving your revised manuscript.

Kind regards,

Nancy Angeline Gnanaselvam

Academic Editor

PLOS Global Public Health

Journal Requirements:

1. We ask that a manuscript source file is provided at Revision. Please upload your manuscript file as a .doc, .docx, .rtf or .tex.

2. If the reviewer comments include a recommendation to cite specific previously published works, please review and evaluate these publications to determine whether they are relevant and should be cited. There is no requirement to cite these works unless the editor has indicated otherwise. 

3. Please review your reference list to ensure that it is complete and correct. If you have cited papers that have been retracted, please include the rationale for doing so in the manuscript text, or remove these references and replace them with relevant current references. Any changes to the reference list should be mentioned in the rebuttal letter that accompanies your revised manuscript. If you need to cite a retracted article, indicate the article’s retracted status in the References list and also include a citation and full reference for the retraction notice.

Additional Editor Comments:

Table 1: There are possible identifiers which can be used to identify the participants. Kindly convert the table to a paragraph by only presenting the summarised findings of demography

The attached transcripts have multiple possible identifiers. Hence I recommend authors to just mention that data is available with investigators and the corresponding author can share the data on reasonable request. Please remove all identifiers such as past employment, age, languages, age of children.

# Author comment: Author response to Decision Letter 1

**DOI:** 10.1371/journal.pgph.0005399.r003

8 Oct 2025

Attachment Submitted filename: Response to Reviewers.docx

# Editor report: Decision Letter 1

**DOI:** 10.1371/journal.pgph.0005399.r004

**Authors:** Nancy Gnanaselvam

17 Oct 2025

Female migrant domestic worker experiences during the COVID-19 pandemic in Hong Kong: A qualitative study

PGPH-D-25-02509R1

Dear Mr. Sumerlin,

We are pleased to inform you that your manuscript 'Female migrant domestic worker experiences during the COVID-19 pandemic in Hong Kong: A qualitative study' has been provisionally accepted for publication in PLOS Global Public Health.

Before your manuscript can be formally accepted you will need to complete some formatting changes, which you will receive in a follow up email. A member of our team will be in touch with a set of requests.

Please note that your manuscript will not be scheduled for publication until you have made the required changes, so a swift response is appreciated.

IMPORTANT: The editorial review process is now complete. PLOS will only permit corrections to spelling, formatting or significant scientific errors from this point onwards. Requests for major changes, or any which affect the scientific understanding of your work, will cause delays to the publication date of your manuscript.

If your institution or institutions have a press office, please notify them about your upcoming paper to help maximize its impact. If they'll be preparing press materials, please inform our press team as soon as possible -- no later than 48 hours after receiving the formal acceptance. Your manuscript will remain under strict press embargo until 2 pm Eastern Time on the date of publication. For more information, please contact globalpubhealth@plos.org.

Thank you again for supporting Open Access publishing; we are looking forward to publishing your work in PLOS Global Public Health.

Best regards,

Nancy Angeline Gnanaselvam

Academic Editor

PLOS Global Public Health
